# A Clustering WSN Routing Protocol Based on k-d Tree Algorithm

**DOI:** 10.3390/s18092899

**Published:** 2018-09-01

**Authors:** John Anzola, Jordán Pascual, Giovanny Tarazona, Rubén González Crespo

**Affiliations:** 1 Department of Electronic Engineering, Fundación Universitaria Los Libertadores, Cr. 16 # 63A-68 Bogotá, Colombia; jpanzolaa@libertadores.edu.co; 2 Department of Engineering, Distrital University Francisco José de Caldas, Cr. 7 # 40B-53 Bogotá, Colombia; gtarazona@gmail.com; 3 Department of Computer Sciences, University of Oviedo, Street San Francisco, 33003 Oviedo, Spain; jordansoy@gmail.com; 4 Technology and Engineering Department of UNIR, Universidad International de la Rioja (UNIR), C/Almansa 101, 28040 Madrid, Spain

**Keywords:** k-d tree algorithm, hierarchical protocol, quality of service, routing protocol, WSN, clustering

## Abstract

Clustering in wireless sensor networks has been widely discussed in the literature as a strategy to reduce power consumption. However, aspects such as cluster formation and cluster head (CH) node assignment strategies have a significant impact on quality of service, as energy savings imply restrictions in application usage and data traffic within the network. Regarding the first aspect, this article proposes a hierarchical routing protocol based on the k-d tree algorithm, taking a partition data structure of the space to organize nodes into clusters. For the second aspect, we propose a reactive mechanism for the formation of CH nodes, with the purpose of improving delay, jitter, and throughput, in contrast with the low-energy adaptive clustering hierarchy/hierarchy-centralized protocol and validating the results through simulation.

## 1. Introduction

Wireless Sensor Networks (WSN) have exploded in popularity in the last few years. Part of this growth is due to the popularization of the Internet of Things (IoT), where connectivity, sensitivity, interaction, and energy are elements of the systems in a WSN. In a WSN, a node is defined as the minimal functional unit of a network and is comprised of a sensor/actuator, a central processing unit (CPU), a memory bank, a wireless transceiver, and a power source. As a unit, the node suffers energy depletion of its internal battery as a result of sensing, processing, data transmission and reception.

Interaction through wireless transmission in a WSN includes issues such as link viability, time to establish communication, data loss due to competition overuse of a wireless channel, data loss due to simultaneous transmission attempts, data loss due to repeated network flooding, and data loss due to transmission range.

Network scalability problems are caused by the birth, reboot, and death of one or several nodes in the network. Link problems in WSNs include neighbor discovery, message, loss, latency, and congestion. WSNs also have routing problems such as communication path and loop discovery [1]. In general lines, WSNs have a wide range of problems, although most of them have been addressed through communication protocols.

The aforementioned problems have been approached through flat and hierarchical routing structures. In a flat routing structure, all the nodes in the network play the same role in end-to-end routing protocols. In hierarchical routing structures, nodes are classified by functionality and the network is divided into groups or clusters, each of which chooses a leading node that is called Cluster Head node (CH). The CH node coordinates activities inside and outside the cluster with non-CH nodes.

The main feature of flat routing structures is the ability to establish communication between any two nodes in the network without the participation of a central node. These networks, also called ad hoc networks, can operate in isolation, without connection to network infrastructure such as the Internet.

A hierarchical routing structure organizes large-scale ad hoc networks into groups or clusters, with the objective of improving network efficiency beyond the attainable level of flat routing structures. Although hierarchical routing can increase control traffic, its topology allows for data traffic to be confined within each cluster. Nodes inside each cluster can organize to optimize communications and reduce interference caused by simultaneous data transmission.

Cluster-based hierarchical routing presents some advantages in scalability and communication efficiency. Protocols based on this schema have been used to achieve routing efficiency in connection to node energy levels. That is, nodes with higher energy reserves are candidates to becoming CH nodes, and those with lower energy levels are used to monitor the environment. In this type of routing, CH nodes have specific functions to improve the scalability, lifetime, and energy efficiency of the network.

Cluster-based routing structures represent a simpler approach to the issues of WSNs, lowering the complexity of flat routing structures [2]. However, cluster-based routing schemes are, in general, comprised of a cluster-formation and a CH-node selection mechanism. The behavior of the latter has an impact on the WSN’s general performance since a high degree of variability in this mechanism creates a proportional variability of the network’s response regarding delay, jitter, and throughput, and an inversely proportional response in energy levels.

The cluster formation mechanism creates clusters of varying sizes and, as a consequence, of varying density. This cluster formation method has an impact on the behavior of the network, which responds according to its cluster formation type. For example, a heterogeneous cluster formation causes data traffic within the clusters to become heterogeneous, that is, having varying dataflow responses in the majority of network nodes. This flow variability causes the network to show different delay and jitter values, limiting the usefulness of resource-intensive applications.

Given the problems found in the cluster formation and CH node selection mechanisms, our goal is to propose a WSN communications protocol that uses a hierarchical routing schema called H-kdtree. Its routing algorithm is based on the k-d tree algorithm, which allows creating partitions in an area with the mean of the data of one of its dimensions. Additionally, the H-kdtree protocol proposes a low-variability CH node generation mechanism, with a positive impact on delay, jitter, and throughput, compared with the low-energy adaptive clustering hierarchy protocol (LEACH) and low-energy adaptive clustering hierarchy-centralized (LEACH-C).

As a comparison, we have selected the LEACH and LEACH-C protocols, on account of being the most widely used hierarchical clustering protocol and also the most discussed in the literature. Clustering protocols work through one or several metrics that provide the necessary ability to manage network traffic efficiently and to improve the experience of a user or machine in different network environments. These improvements are usually in one or several network metrics such as load balancing, energy consumption, scalability, latency reduction, data traffic maximization, errors and data loss minimization. Quality of Service (QoS) is the improvement in one or several of these network metrics.

This article analyzes the performance of the LEACH, LEACH-C and the proposed H-kdtree protocol, by measuring the following metrics: delay, jitter, throughput, packet drop rate (PDR), and average network energy.

This article is structured as follows: Section 2 includes a review of literature related to cluster formation and CH node selection. The fundamental basis for LEACH, LEACH-C and k-d tree is described in Section 3. Protocol considerations and a description of the configuration and data transmission phases are discussed in Section 4. Section 5 includes parameters, metrics, and results of the simulation of the proposed protocol. Finally, Section 6 presents the conclusions.

## 2. Related Work

This section presents some of the most relevant works about techniques and mechanisms for cluster formation and CH node selection.

### 2.1. Cluster Formation

In WSNs, cluster formation is a technique that allows the classification of nodes in groups or clusters so that every node in a cluster shares a certain degree of homogeneity regarding the techniques, rules or heuristics on which the selection is based.

In the majority of distance-based cluster formation algorithms for WSNs, it is assumed that the nodes of the network know their a priori location in the plane. However, the literature includes proposals where nodes transmit their distance to a Base Station node (BS) via either Received Signal Strength Indicator (RSSI) [3] or geo-positioning by using the Global Positioning System (GPS) [4].

The main objective of partition clustering algorithms in WSNs is to divide the nodes into k partitions, according to their position. Each partition is considered a cluster. The partitioning technique will depend on an objective function. The most representative clustering mechanisms employed in WSNs are k-means [5,6,7], fuzzy c-means [8], k-medoids [4,9].

The hierarchical grouping method in a WSN tries to build a tree-based network topology mainly derived from the position of each node. This grouping process is represented by topologies with two or more jumps to a BS node. Some of the most representative algorithms are LEACH [10], LEACH variations [11,12,13].

Density-based cluster formation methods assume that nodes in each group are extracted from a probability distribution in relation to the total number of nodes in the network. Some works in this area are: to maximize the network lifetime [14,15], adaptive clustering [16], and density-based fuzzy imperialist competitive clustering algorithm [17]. The approaches of cluster formation based on cooperation have provided solutions to the problem of energy management using the information of the energy spectrum detected [18].

### 2.2. Cluster Head Selection

Cluster-head capabilities depend on the clustering objectives or focus used in their formation, and taking into account the capabilities of the nodes and their effective range. Most of the work on CH node selection has focused on the energy capacity of network nodes [19,20]. The following node attributes have been differentiating factors among the various clustering and CH node selection schemes.

Regarding the mobility or stationarity of nodes and CH nodes in WSN hierarchical routing, some proposals adhere to mobile nodes where membership to each node changes dynamically and clusters are required to auto-configure and keep an updated members list with incoming and outgoing nodes [21,22]. Other proposals favor a stationary approach, in which all nodes tend to group into stable clusters, which allows for simpler network administration and intra/inter-cluster communications [23].

The literature includes types of nodes of extended hardware capability [24]. Some algorithms include references to advanced nodes, defined as those nodes with more energy [25]. Other approaches focus on network interoperability, with nodes for different types of connectivity [26], and interoperability between nodes of fixed and wireless networks [27].

The types of roles of a node in a hierarchical protocol can be either a data transmission node or a CH node, and each node can change roles between rounds. Some proposals include auxiliary nodes that can take the role of the CH node in cases where the CH node fails [28]. Other approaches use a fuzzy inference system to improve the adaptability of the selection of CH nodes, finding that the stochastic selection methods can not guarantee the availability of CH nodes [29].

This article takes into account several aspects that are not approached completely in the cited literature. In particular, this article proposes a hierarchical routing protocol based on the k-d tree algorithm and on a reactive mechanism for the formation of CH nodes, validating its QoS through an experimental approach through simulation. Complementary information can be consulted in [4,30], also aspects related to the location of nodes in WSN [31], design problems [32] and the extension of possible applications in other areas of knowledge such as robotics [33,34], social networks [35] and applications that can support QoS [36,37].

## 3. Background and Preliminaries

The following subsections present the working principles of the LEACH and H-kdtree algorithms, as their key concepts are used in the proposed protocol.

### 3.1. The LEACH Protocol

**Figure 1:**
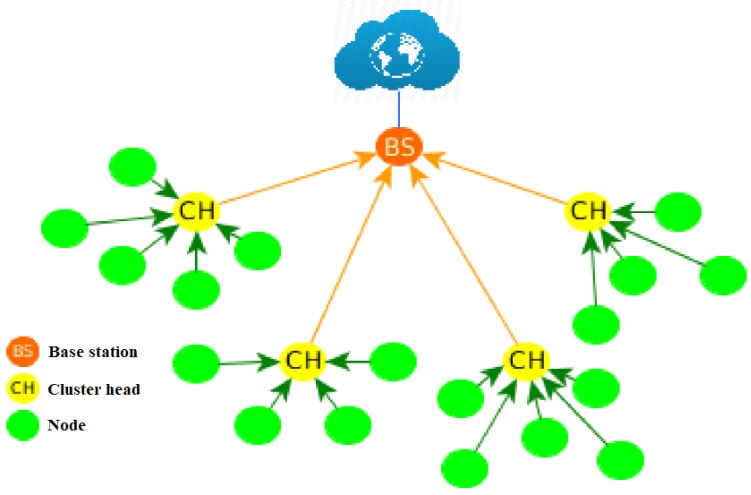
LEACH protocol topology.

The Low-Energy Adaptive Clustering Hierarchy protocol (LEACH) uses a cluster-based routing scheme to minimize total network energy consumption. In LEACH, nodes deployed in an area are organized into clusters, with each cluster having a CH node, as shown in Figure 1. The communication process is divided into two phases: the configuration phase and the stable state phase [10].

**Figure 2:**
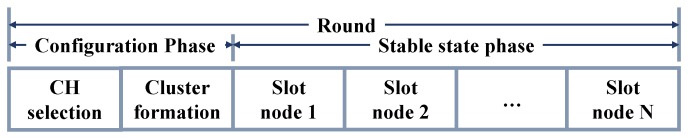
LEACH protocol phases.

During the configuration phase, the CH nodes and member nodes of each cluster are selected. During the stable state phase, nodes are added to the CH node and remain in waiting to initiate data transmission to the Base Station (BS) node, using the sequence shown in Figure 2. In the literature, many authors refer to the base node as Sink node [38], which we refer to from this point on as Sink/BS node. The duration of the stable state phase is longer than the configuration phase, due to the processing work performed in it. Consequently, energy consumption during the stable state phase is higher.

During the configuration phase, the nodes that will become CH nodes for the current round are selected independently and randomly, with the requirement that their energy is greater than zero and a lower threshold. The capacity to become a CH node is determined by the generation of a random number $\left(R_{n}\right)$ where $R_{n}\in \left[0,1\right]$. The CH node is selected if $R_{n}\leq T\left(n\right)$, where T(n) is a threshold value obtained from Equation (1). The CH node will transmit its nomination to the nodes in the cluster. The nodes in the cluster are selected by their distance to the CH node and, in some cases, this is measured through the received signal strength indicator (RSSI) [39]:

(1) $$t\left(n\right)=\left\{\begin{array}{cc} \frac{p}{1-p\cdot (r\cdot mod\cdot \frac{1}{p}))}, & \mathrm{if}\;n\in G,\\ 0, & \mathrm{another}\;\;\mathrm{case}. \end{array}\right.$$

where *p* is the probability for a node to become CH node over all the nodes in the network, *r* is the current number of selection rounds, and *G* is the set of nodes that were not selected as CH nodes before $\frac{1}{p}$ rounds.

The CH nodes selected for each cluster will reserve a free slot for data transmission of the member nodes by using a distribution of a TDMA programming table that will indicate each CH node the data transmission sequence, giving CH nodes the possibility of staying in repose for the longest possible time.

Using TDMA for data transmission prevents collisions within each cluster [40]. In the steady state phase, CH nodes compile data from the nodes in each cluster and send them to the Sink/BS node. Data redundancy may occur during this phase. The redundant packets being processed and transmitted to the Sink/BS node result in an increase in unnecessary network traffic and overall network bandwidth, which directly affects energy consumption. The more redundant data is processed, the more energy will be wasted. Data transmission to CH nodes saves energy in comparison to direct data transmission from the nodes to the Sink/BS node. Therefore, to avoid the early death of CH nodes, all the nodes in the network will elect other CH nodes, repeating both phases for every round during the lifetime of the network.

**Figure 3:**
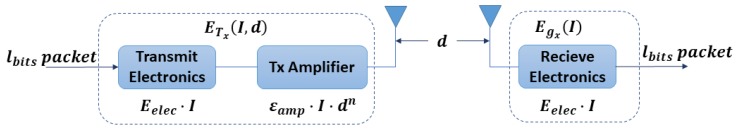
Radio energy model.

For every node, we used the energy model described in [41], which is shown in Figure 3. This assumes that the wireless channel is completely symmetrical, so the energy used in transmitting a message through the round-trip route between a network node and the Sink/BS node through the CH node, $v_{i}$ and $v_{j}$ is equal. The free space channel model used is $d^{2}\left(v_{i},v_{j}\right)$, as the nodes are located in a plane and remain static and we assume that there is a direct line of sight between the network nodes and the Sink/BS node. If the communication distance $d(v_{i},v_{j})$ from $v_{i}$ to $v_{j}$ is greater than the threshold distance $d_{0}$, that is, $d\left(v_{i},v_{j}\right)\geq d_{0}$, the model chosen is the multipath fading model $d^{4}\left(v_{i},v_{j}\right)$. This is the case for both the LEACH protocol and the H-kdtree protocol presented in this article. Energy consumption $\left(E_{T}\;l,d\left(v_{i},v_{j}\right)\right)$ is calculated as:

(2) $$E_{T}=\left\{\begin{array}{c} l\cdot E_{elec}+l\cdot \epsilon_{fs}\cdot d^{2}\left(v_{i},v_{j}\right),\;d\left(v_{i},v_{j}\right)<d_{0},\\ l\cdot E_{elec}+l\cdot \epsilon_{amp}\cdot d^{4}\left(v_{i},v_{j}\right),\;d\left(v_{i},v_{j}\right)\geq d_{0}, \end{array}\right.$$

where *l* is a message of $l_{bits}$ to be transmitted over $d(v_{i},v_{j})$, given by $E_{R}\left(l\right)=l\cdot E_{elec}$, where $E_{elec}$ represents the loss of the transmission circuit as a function of digital encoding, the type of modulation used, filtering processes and signal dispersion, based on energy coefficients $\epsilon_{fs}\cdot d^{2}\left(v_{i},v_{j}\right)$ and $\epsilon_{amp}\cdot d^{4}\left(v_{i},v_{j}\right)$ for power amplifiers in two channel models.

One of the characteristics of LEACH and its different variations is that it maintains the configuration phase, transmission and a hierarchical topology of two jumps. Under these characteristics, the algorithm LEACH runs in O(n·log·m) time for *n* sensor nodes and *m* CHs [42].

### 3.2. LEACH-C Protocol

The LEACH-C protocol is a centralized version of the LEACH protocol, and, therefore, it uses the same phases of the LEACH protocol, which are configuration and transmission phase forming rounds. During the LEACH-C configuration phase, each node in the network sends a packet to the Sink/BS node that contains the location and power level. The Sink/BS node calculates the average energy value of all the nodes, selected as possible node CH, only the nodes with more energy than the average value of the energy of all the nodes in the network.

The Sink/BS node uses an annealing algorithm for the formation of clusters [43,44]. The other LEACH-C operations are the same as those of LEACH and the results show that LEACH-C has energy improvements on LEACH [45], for the following reasons:

- The Sink/BS node is static and is the one that organizes. the roles of each node in the network, centralizing information and cluster formation.
- When clusters are formed, they do not communicate between nodes to save energy.
- The Sink/BS node in the configuration phase establishes the CH nodes beforehand; therefore, the network can use energy more effectively.

### 3.3. k-d Tree Algorithm

**Figure 4:**
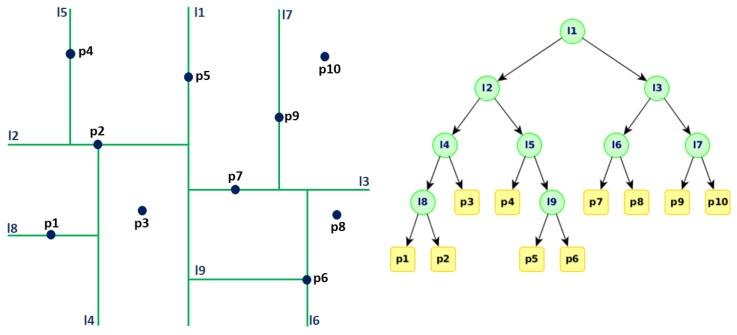
Visualization of the k-d tree algorithm.

Given the problem of two-dimensional rectangular range queries, a rectangle is divided into a smaller rectangle, which in turn is divided into another rectangle, and the process is repeated *n* times (depth), obtaining successive smaller areas. Within these areas (rectangles), we can locate a set of points that can be referenced as a unit, accessible via a route originated in a binary tree. Figure 4 shows the basic working idea of the k-d algorithm [46].

Given *P* as the set of *n* points in the plane, we can assume as a principle that no two points share the same (x,y) coordinates. There are no cases where two or more points are superimposed.

**Definition** **1.** *In a two-dimensional rectangular range query in P, we ask for the points in P inside the query rectangle $\left[x:x^{\prime }\right]*\left[y,y^{\prime }\right]$. A point $p:=(p_{x},p_{y})$ is inside that rectangle if and only if:*

(3) $$p_{x}\in \left[x:x^{\prime }\right]\;and\;p_{y}\in \left[y,y^{\prime }\right].$$

**Definition** **2.** *Recursive binary search tree for a set of points in one dimension is divided into two subsets of approximately the same size, based on the median of the set of points. In this way, the root contains two subsets distributed in two subtrees, where each subset of the subsets already created become subtrees that will be processed recursively.*

The procedure to build the k-d tree has two parameters: one set of points *P* and an integer value that represents the depth of the subtree root, as shown in Algorithm 1.
**Algorithm 1** k-d tree algorithm.**Require:** A set of points (x,y) named *P* and the tree depth value **Ensure:** The root of the k-d tree1:**function**: *build_k-d_tree*(P,depth)2:**if** (*P* contains only one point) **then**3: **return** the root with that point4:**else if** (depth is an even number) **then**5: divide the values of *P* into two subsets by using the median of the *x* coordinates of the *P* set. It generates two subsets:6: **if** $\left(P_{x_{i}}<\;median\left(x\right)\right)$ **then**7:  $P_{x_{i}}^{1}:=subset_{left}$8: **else**9:  $P_{x_{i}}^{2}:=subset_{right}$10: **end if**11:**else**12: divide the values of *P* into two subsets by using the median of the *y* coordinates of the *P* set. It generates two subsets:13:**end if**14:depth=depth+115:**end function**

For the function of the Algorithm 1:

- Creates a root node with two subsets $P_{{\left(*\right)}_{i}}^{1}$ for the left side of the tree and $P_{{\left(*\right)}_{i}}^{2}$ for the right side of the tree.
- (∗) is the subset of initial data where the partitioning of points will begin. In this algorithm, *x* is used for even depth values and *y* is used for odd values.
- Repeat steps 1 to 14 to create the branches of the tree, where the input parameter with the input data set is the subset $P_{{\left(*\right)}_{i}}^{1,2}$.

The time used in *k* groups of the kd-tree algorithm is O(k) for the depth of the tree and because *P* is a finite set each partition has a length in time of O(log(n)), so the time total used in the algorithm is O(log(n)+k) [46].

## 4. Proposed Protocol

This section describes in detail the hierarchical k-dimensional tree algorithm (H-kdtree). Unlike conventional WSN routing protocols like LEACH, HEED, TEEN, etc. [47,48], which used a few variations on Equation (1) to form clusters and select CH nodes according to nodes’ residual energy and their distance to the Sink/BS node, H-kdtree uses the one-dimensional clustering principle taken from the k-d tree algorithm. This algorithm generates a hierarchical two-hop network topology similar to LEACH’s.

Next, we will explain the clustering mechanism of the k-d tree algorithm intuitively, using the data from Table 1.

**Figure 5:**
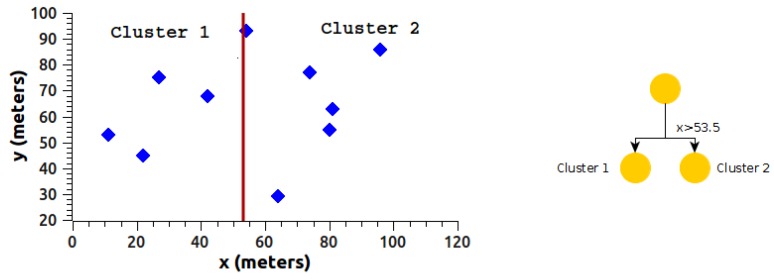
k-d tree algorithm with *x*-dimension.

The data in Table 1 are divided into two clusters, starting data partition with data from the *x* dimension, implementing the median value ($v_{m}$), where $v_{m}=\left(v_{max}+v_{min}\right)/2$. For the data in Table 1, we have a median value of 53.5 for the *x* dimension, as shown in Figure 5.

In Figure 5, the algorithm shows the formation of two clusters. Each of the clusters found contains three variables:

- Dimension used for the division (*x* or *y*),
- Median value ($v_{m}$),
- Limits of the nodes in each cluster.

**Figure 6:**
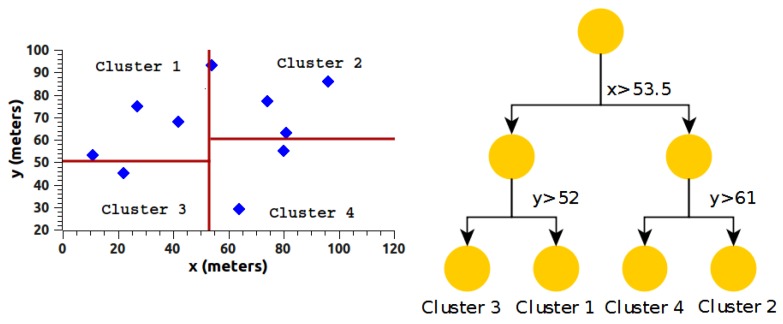
k-d tree algorithm with dimension on *y*.

The limits on the *y* dimension for $cluster_{1}$ and $cluster_{2}$ are, respectively, $29\leq y_{\left(cluster_{1}\right)}\leq 75$ y $29\leq y_{\left(cluster_{2}\right)}\leq 93$. The structure in Figure 5 is divided in the same way but alternating the dimension, which in this case would be y, obtaining a new structure with four clusters, as shown in Figure 6.

**Figure 7:**
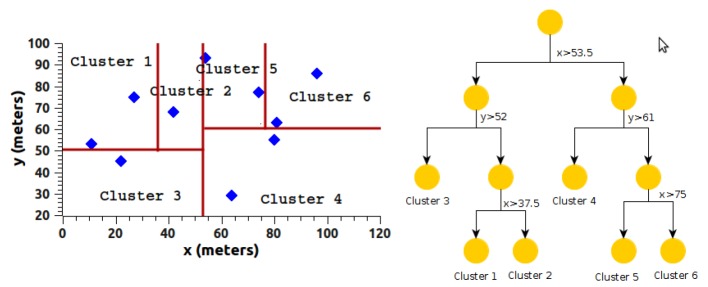
Clustering using the k-d tree algorithm.

Based on the structure obtained so far, as shown in Figure 6, we change dimension and begin to create new partitions, as shown in Figure 7. The process shown so far is repeated iteratively until the stop condition is met, being the number of clusters or the minimum group condition.

Algorithm 2 has the *k* variable as the input parameter. The *k* variable represents the number of clusters desired (this is a parameter similar to the Algorithm 2 has the *k* variable as the input parameter. The *k* variable represents the number of clusters desired (this is a parameter similar to the *k-means* algorithm). If k=3, the first iteration obtains the data in Figure 5. Up to this point, we have two clusters. In the next iteration, we would obtain four clusters, but since the goal is to obtain three clusters, we take the clusters obtained so far and we select the cluster with the highest node count and partition only this cluster. In this way, we obtain the desired three clusters. In case the two clusters obtained in the first iteration have an equal number of nodes, one of them is chosen randomly.

### 4.1. Protocol Considerations

The maximum number of clusters that can be obtained is the total number of nodes divided by four. The minimum cluster condition regarding the number of nodes is three nodes and a CH node. In case we have a remainder smaller than four, the remaining nodes are added to the nearest cluster. In other words, the last partition of the current dimension in the algorithm is not performed.

The working principle of the H-kdtree protocol determines the following condition and consideration for the management of the complete network: Every action in the network is centralized and managed by the Sink/BS node, and all nodes in the network are within the range of the Sink/BS node.

Every node has a minimum energy threshold. This threshold is a function of the power supply voltage of each node. Every node will send the Sink/BS node a “Death” message when their power reserves reach 3% of the minimum operational threshold, informing the Sink/BS node that it is dead.

### 4.2. Configuration Phase

The Sink/BS node begins flooding the network with a broadcasted “Hello” message, to which every node in the network will reply with an acknowledgment message informing their nd energy level.

With the information obtained in the flooding process, H-kdtree begins the cluster formation process, based on the k-d tree algorithm. Once the clusters are formed, the following step is to select the CH nodes, based on the energy levels obtained during the flooding process. The node with the highest energy is selected as CH node. If two or more nodes have the same energy level, one of them is randomly selected to become a CH node.

At this point, the Sink/BS node already has information of every cluster, with their respective CH node and member nodes. The next step is configuring static routes. The Sink/BS node sends the CH nodes the static routes information, which is then forwarded to the rest of the nodes in each cluster. The result is the typical LEACH hierarchical routing, using a two-hop topology.

### 4.3. Transmission Phase

The data transmission phase is divided into rounds. Every round has a time slot of N-nodes, where N-nodes is the number of non-CH nodes in the network. During this period, the Sink/BS node sends a “Request” packet to the first CH node. Once the CH node receives this “Request” packet, it organizes a programmed transmission with the nodes in its cluster by using TDMA as access method. This process is repeated for all the clusters in the network.

**Figure 8:**
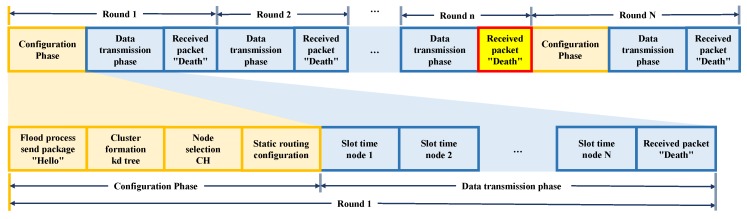
Protocol header H-kdtree.

The “Death” packet informs the Sink/BS node that a node has just died. Nodes in a cluster transmit the “Death” packet to the Sink/BS node using their respective time slot. If a CH node dies, it transmits its “Death” packet to the Sink/BS node when queried by the Sink/BS node. At the end of each round, the Sink/BS node reviews which nodes sent a “Death” packet. In case a “Death” packet arrives at the end of a round, the Sink/BS node begins the configuration phase, as shown in Figure 8.

**Figure 9:**
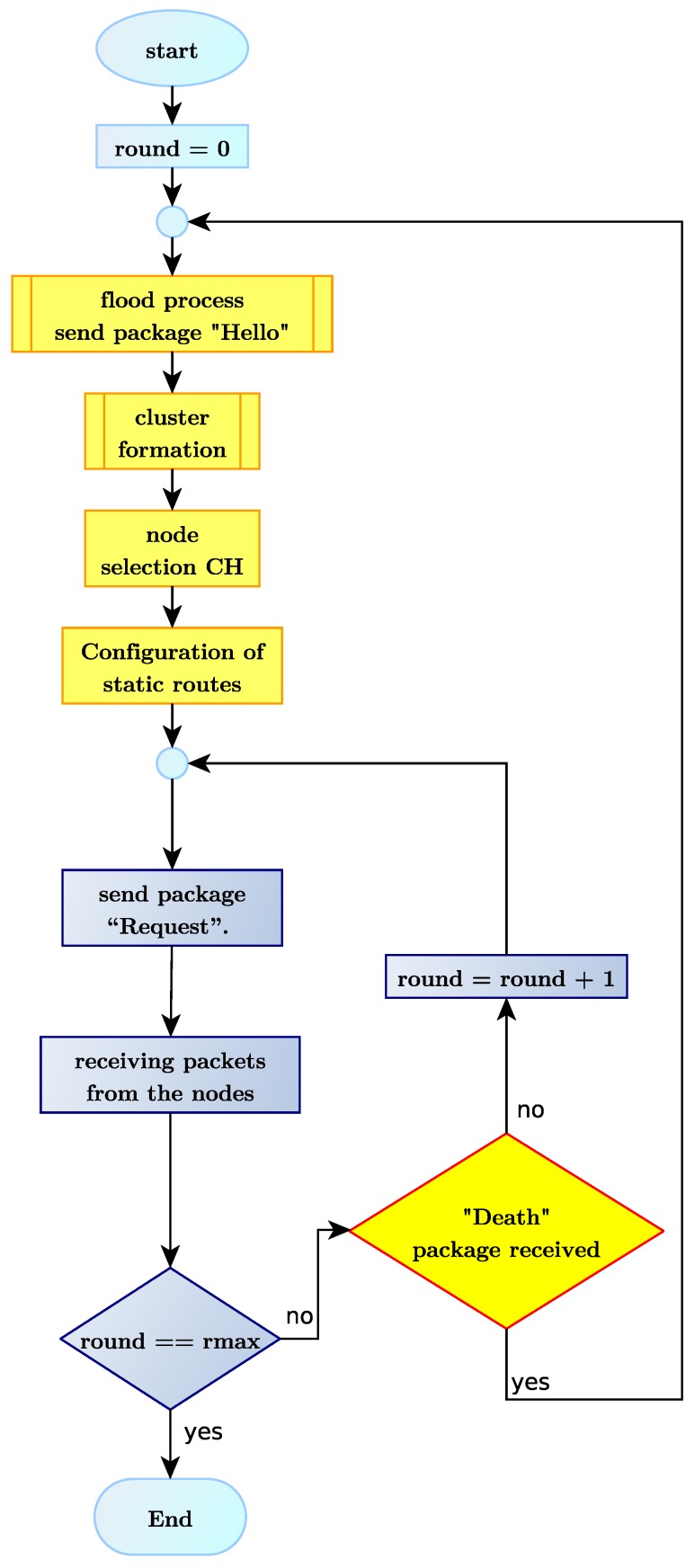
Routing flowchart H-kdtree protocol.

Figure 9 below shows the H-kdtree protocol’s algorithm. Algorithm 2 shows the cluster formation process as a complement to the processes described in this section.
**Algorithm 2** Cluster formation based on the k-d tree algorithm.**Require:** Matrix obtained in the flooding process, with the following fields: $id_{node}$, $x_{cor}$, $y_{cor}$, energy.  The dim variable is the value of the column to that corresponds to the dimension to be selected,  where $x_{cor}=2$, $y_{cor}=3$. **Ensure:** List with the vectors of the positions selected in each cluster1:**function**: *cluster_formation*(mat,dim)#Variable $vec_{1}$, stores the positional values of $cluster_{1}$2:$vec_{1}=rep\left(NA,length\left(mat\left[,1\right]\right)\right)$#Variable $vec_{2}$, stores the positional values of $cluster_{2}$3:$vec_{2}=rep\left(NA,length\left(mat\left[,1\right]\right)\right)$#Calculate the median of the selected partition4:$v_{m}=median\left(mat\left[,dim\right]\right)$#Traverse the data in the selected dimension5:**for** (i in 1:length(mat[,1])) **do**6: *#Subdivide the dimension into two clusters, depending on the median value*7: **if** $\left(mat\left[i,dim\right]>v_{m}\right)$ **then**7:  $vec_{1}\left[i\right]=i$9: **else**10:  $vec_{2}\left[i\right]=i$11: **end if**12: **end for**13:**refturn**$\left(list\left(vec_{1},vec_{2},v_{m}\right)\right)$

## 5. Simulation and Results Analysis

For the simulation, we used NS-2 version 2.35, simulating LEACH, LEACH-C and H-kdtree in the same network environment to make a comparison and obtain metrics in the same simulator. The LEACH, LEACH-C and H-kdtree algorithms were implemented using R version 3.4.3. Implementing the algorithms using R allowed us to generate scripts in “.tcl”, which were embedded in the main script to configure position and initial energy of the nodes, static routing between nodes, CH nodes and Sink/BS node, along with traffic generated in each time slot, and planned information transmission in TDMA.

### 5.1. Simulation Parameters

**Figure 10:**
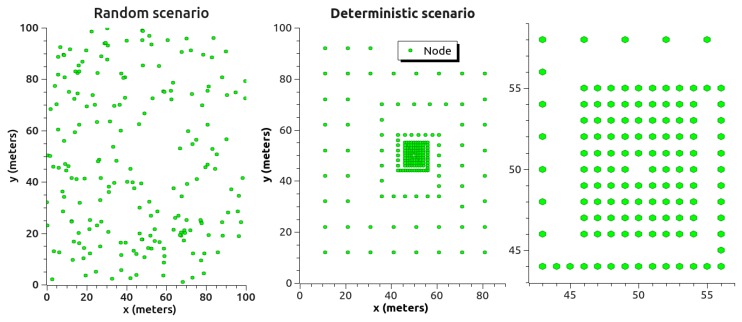
Evaluated scenarios.

There were two simulation scenarios for LEACH, LEACH-C and H-kdtree. Scenario 1 consisted of a random deployment of sensors. Scenario 2 was a sensor deployment with higher density in the central zone of a deterministic scenario, as shown in Figure 10.

The use of random and deterministic node deployment scenarios aimed at abstracting network traffic behavior to evaluate QoS. Node deployment was done in a 100 m × 100 m area, maintaining the same density in both scenarios. The deterministic scenario aimed to evaluate network traffic in a scenario with higher density in its central area, with the objective of analyzing the influence of clustering in both types of scenarios.

Table 2 shows the simulation parameters used. These parameters are used in literature mainly to evaluate the performance of LEACH and LEACH-C [49,50,51,52].

Hierarchical protocols present two types of networks, according to their energy: homogeneous and heterogeneous networks. In homogeneous networks, the initial energy level is the same for all the nodes in the network. In heterogeneous networks, the nodes in the network have different initial energy values.

In the scenarios shown in Figure 10, the network is divided into two energy levels. This energy division is represented by the parameter *m*, which is used to calculate cluster energy $\left(E_{cls}\right)$[53]. $\left(E_{cls}\right)$ can be calculated as follows:

(4) $$E_{cls}=N_{cls}\cdot E_{0}\cdot \left(1-m\right)+N_{cls}\cdot m\cdot E_{0}\cdot \left(1+\alpha \right),$$

where $E_{0}$ is the initial energy of a regular node, $N_{cls}$ is the number of clusters, and *m* is the percentage of nodes in the network with an advanced energy level. The quantity of advanced energy is represented by α. H-kdtree uses the simulation parameter *k* to obtain the depth with which the nodes in the network will be partitioned. This partition is similar to LEACH’s *p* parameter, which is used to estimate the expected number of CH nodes.

### 5.2. Simulation Metrics

To assess data traffic performance and QoS in the proposed scenarios, we used the following performance metrics.

#### 5.2.1. End-to-End Delay (EED)

It is the time elapsed since a packet is sent by a node and until the packet is received by the Sink/BS node, taking into account the latencies experienced in all its path, including the latency of the CH node [54]. It is calculated as follows:

(5) $$EED=T_{rec}-T_{sent},$$

where $T_{rec}$ is the time when the Sink/BS node receives a data packet, and $T_{sent}$ is the time when a non-CH node sends that data packet.

#### 5.2.2. Throughput

This is the number of bits that can be transmitted by each node to the Sink/BS node in a period of time [55]. The sum of the throughput of each node in the network is known as network throughput. The throughput is obtained by dividing the total number of packets received (by the Sink/BS node) by the total time for each round

(6) $$Throughput=\frac{packets_{received}\times packet_{size}}{total\_time_{transmitted}}.$$

#### 5.2.3. Packet Delivery Ratio (PDR)

This is the ratio between the number of data packets received by the Sink/BS node and the number of data packets sent by the network nodes [56]. The PDR value can be obtained by the following equation:

(7) $$PDR\left(\%\right)=\frac{\sum No\_packet_{received}}{\sum No\_packet_{sent}}\times 100.$$

#### 5.2.4. Jitter

Jitter can estimate the instability of a communication link. It is the variability in the time needed by a packet to reach the previously transmitted packet [57]. It is calculated by:

(8) $$Jitter=\sum_{i=0}^{1}\frac{\sqrt{Delay_{i}-\bar{Delay}}}{N}.$$

#### 5.2.5. Auxiliary Metrics

Other performance metrics used in hierarchical routing protocols are summarized below. These metrics are the synthesis of the results in terms of node extinction per round. The metrics evaluated in both protocols are:

- First node died (FND): It is the number of rounds in the network until the first node has depleted its energy and died.
- Half of nodes died (HND):It is the number of rounds in the network until half of the nodes in the network have depleted their energy and died.
- Last node died (LND): It is the number of rounds in the network until all nodes in the network have depleted their energy and died.

### 5.3. Results and Discussion

**Figure 11:**
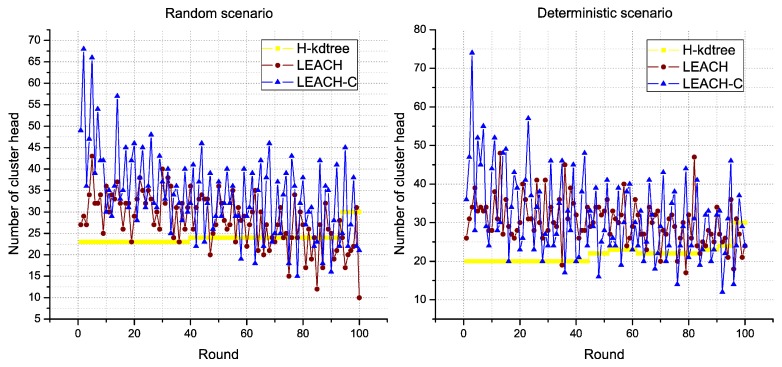
Cluster Head formation behavior.

In the results obtained, one of the most stable parameters found in the proposed H-kdtree protocol is related to the formation of CH nodes, as shown in Figure 11. In this section, we analyze the impact of low variability in CH node formation, in relation to the following performance metrics: delay, throughput, and jitter, and their results are interpreted as QoS.

**Figure 12:**
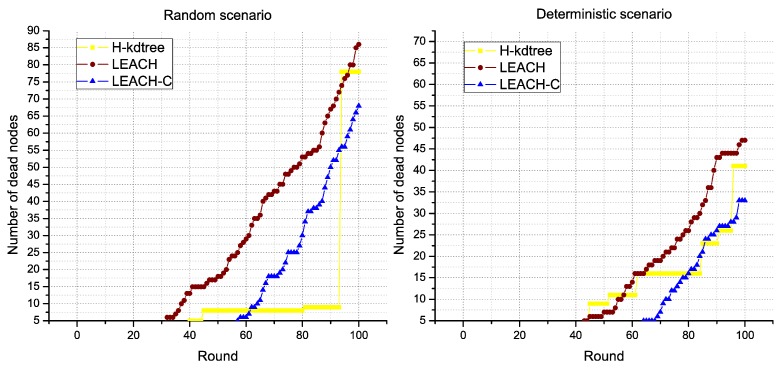
Dead nodes.

Regarding CH node formation in each round, we observed that LEACH and LEACH-C reduces the formation of CH nodes as nodes die. On the contrary, H-kdtree increases CH node formation in the network because of the minimum nodes per cluster value: as the number of nodes goes down, H-kdtree tends to maintain its *k* value by iterating more times, which tends to comply with the minimum nodes per cluster condition. This behavior can be seen in Figure 11 and Figure 12 after round 80.

**Figure 13:**
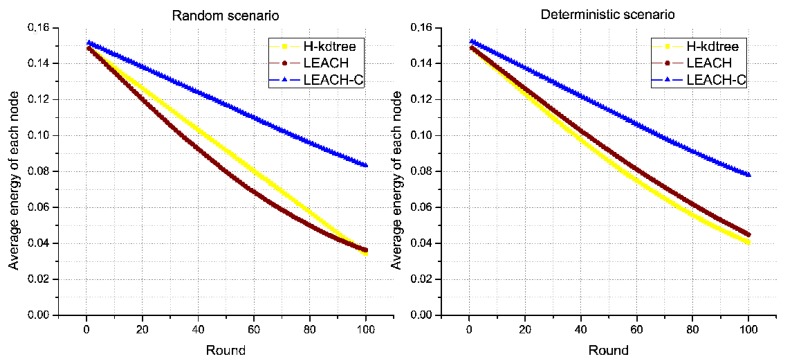
Average energy.

Regarding energy levels, we did not find a significant variation or tendency in H-kdtree compared to LEACH and LEACH-C, as shown in Figure 13. The reason is that the energy that LEACH and LEACH-C used in node formation is offset by the energy used in selecting CH nodes in H-kdtree, being that the latter is more stable in terms of variations and allows for a more stable behavior in the data transmission phase. Figure 12 shows node death compared to energy. H-kdtree resulted in a lower number of dead nodes in both scenarios, compared to LEACH and LEACH-C.

The scenarios allow us to assess QoS features from a hierarchical point of view for LEACH, LEACH-C and H-kdtree, as the protocols share a clustering topology with a two-hop distance to the Sink/BS node.

Regarding delay, the scenario with random node deployment shows more delay than the deterministic scenario with more density in its central area. H-kdtree maintains a stable number of CH nodes for the maximum possible number of rounds. This simplifies the work of the Sink/BS node, as each node has an identifiable death threshold that, when reached, triggers the transmission of a “Death” packet to the Sink/BS node to inform its death. This feature enables H-kdtree to maintain a network topology for the maximum possible number of rounds. This is not possible for LEACH and LEACH-C, per changes in the network topology in each round.

In the rounds we evaluated in the random scenario, H-kdtree changes the topology of the network five times, and nine times in the deterministic scenario, as shown in Figure 12. LEACH and LEACH-C changed its topology 100 times in both scenarios (in each round, the topology changes).

CH nodes do not transmit sensory data. CH nodes compile packets from each cluster and retransmit them to the Sink/BS node. For this reason, if the network topology remains constant, delay, jitter, and throughput metrics will not vary, as these three metrics are a function of time. On the other hand, TDMA divides the network nodes into tie slots and ensures no packet loss due to simultaneous transmission.

**Figure 14:**
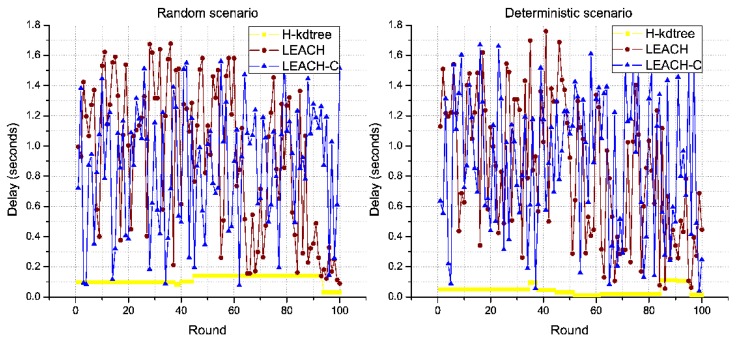
Delay behavior.

**Figure 15:**
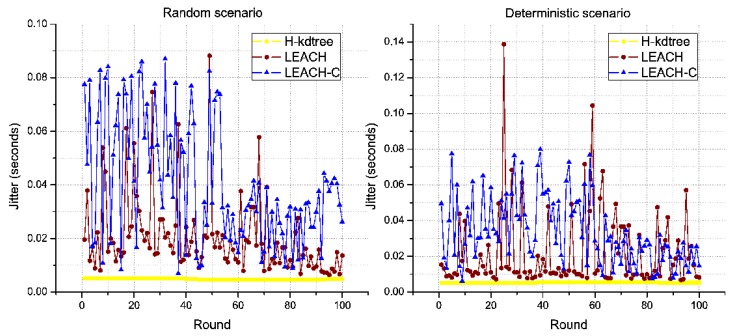
Jitter behavior.

In both scenarios, H-kdtree shows the lowest values for Delay and Jitter, due to H-kdtree’s low variability of topology, compared to LEACH and LEACH-C. This is shown in Figure 14 and Figure 15.

**Figure 16:**
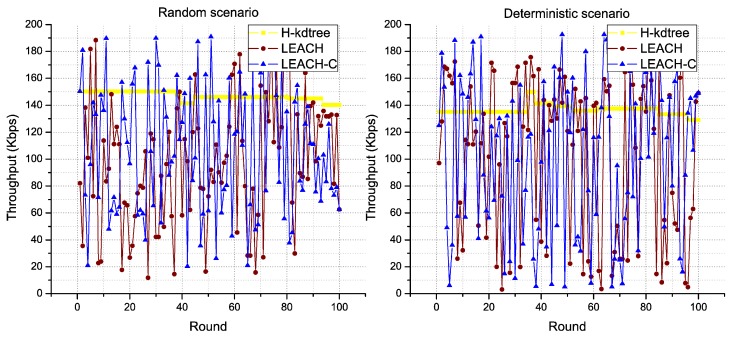
Throughput behavior.

The stability of H-kdtree allows data traffic to remain constant, with very low variability in the rounds we evaluated as compared to LEACH and LEACH-C, in both scenarios. The results shown in Figure 14, Figure 15 and Figure 16 support our recommendation for multimedia applications, due to its stability in delay, jitter, and throughput metrics.

### 5.4. Results Summary

**Figure 17:**
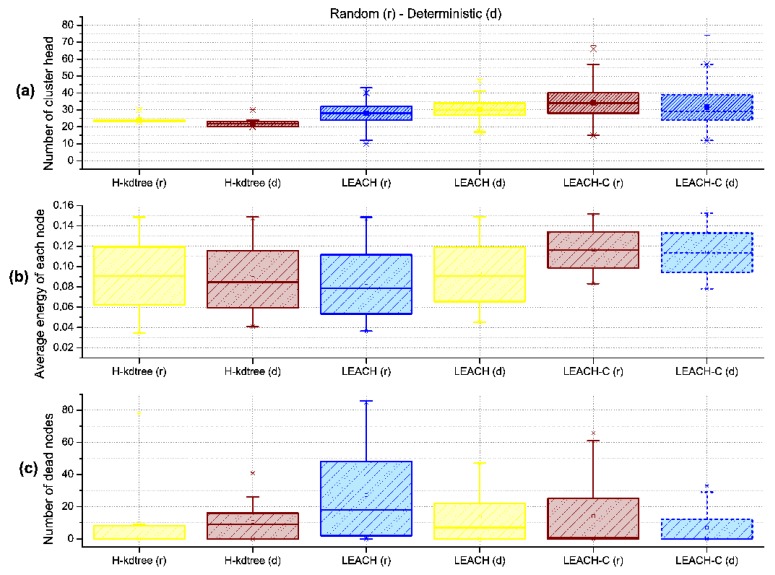
General energy metrics with 200 nodes.

Figure 17 shows a general overview of the distribution symmetry of metrics, showing that (a) LEACH and LEACH-C shows a tendency to a symmetrical distribution of CH nodes in both the random and deterministic scenarios (This is due to the random function used in its algorithm). This result does not occur with H-kdtree. The four anomalous values shown in LEACH and LEACH-C are the minimum and maximum values in the observed rounds, outside the first and third quartile.

The anomalous values for H-kdtree in the deterministic scenario are in the last rounds. Regarding CH node formation, H-kdtree shows low variability, with most of the data in the first and second quartile. Our interpretation is that at least 75% of CH nodes formed showed low variability along the observed rounds.

Figure 17a and Table 3 show the low variability in the number of CH nodes. H-kdtree kept the number of CH nodes below the number of CH nodes generated by LEACH for the first 75 rounds (Q3). H-kdtree showed a reduction of 14.21% for the random scenario and of 30.14% for the deterministic scenario, as compared to LEACH.

Regarding average energy consumption for all nodes as shown in Figure 17b and Table 4, the energy behavior was similar in both H-kdtree and LEACH during the observed rounds. LEACH-C presents an energy efficiency above 40%, in relation to LEACH and H-kdtree. Figure 17c and Table 5 shows a proportional relationship between CH node formation and the number of dead nodes along the observed rounds.

To compare the results and quantify them as percentages, we used normalized averages. This will allow us to estimate the performance improvement for the metrics in the protocols, taking as a reference point the results for LEACH in the random scenario.

Figure 17c and Table 5 shows that between quartiles Q1 and Q3, which correspond to the 25% and 7% of the number of observed rounds, H-kdtree presented the lowest number of dead nodes. In its final stage, H-kdtree shows the highest number of dead nodes due to the on-demand CH node generation mechanism, compensated with energy expenditure after the Q3 quartile. Although H-kdtree does not provide an optimal node energy distribution, the on-demand CH node selection mechanism provides a significant improvement in QoS, as measured in delay, jitter, and throughput.

**Figure 18:**
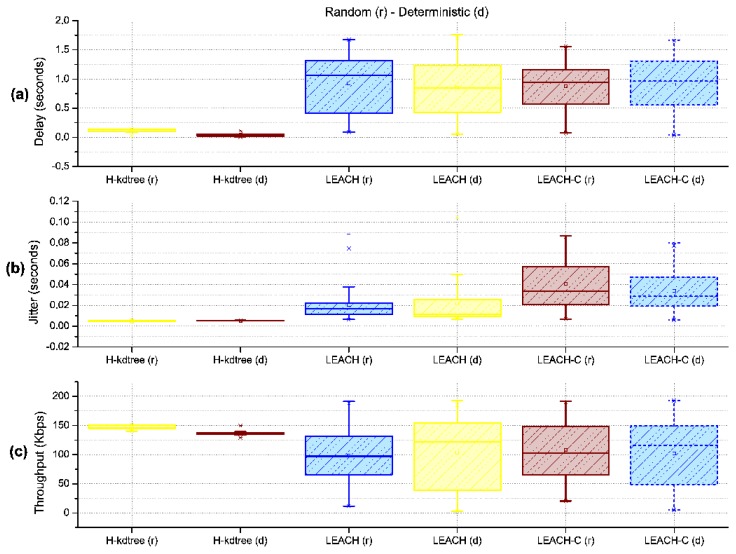
General QoS metrics.

The variability in delay, jitter, and throughput of the proposed H-kdtree protocol as compared to LEACH and LEACH-C is very low in the random and deterministic scenarios, as shown in Figure 18. This is because the level of dispersion of values from the central trend in LEACH and LEACH-C is quite noticeable. These observations allow us to estimate QoS both quantitatively and qualitatively, supporting applications with higher demands.

With the level of delay variability shown in Figure 18a and Table 6, H-kdtree is able to provide QoS in networks with hierarchical topologies. In both the random and deterministic scenarios, H-kdtree showed a reduction in delay of 87.72% in the random scenario and of 95.39% in the deterministic scenario, compared to LEACH. With respect to LEACH-C, H-kdtree presented a reduction of 82.095% for the random scenario and 93.1% for the deterministic scenario.

Jitter response shown in Figure 18b and Table 7, interpreted as temporal variability in packet transmission, is also due to using TDMA for medium access. The level of jitter reduction found in the random scenario was of 76.52%, and 74.4% for the deterministic scenario. The values for delay, jitter, and variance were so low during the observed rounds that we can estimate that H-kdtree can guarantee the requirements for multimedia applications. The reason for this is that H-kdtree’s on-demand CH node selection mechanism is able to manage WSN resources efficiently.

The results obtained show that H-kdtree is able to provide QoS in applications with high restrictions in bandwidth and delay, at the expense of energy consumption. On the other hand, LEACH and LEACH-C are able to adapt to energy fluctuations in the network but is not capable of supporting multimedia applications or time restrictions, on account of its high variability.

Regarding bandwidth and the amount of data that it can transport per round, H-kdtree showed an increase of 48.96% in the random scenario and of 39.37% in the deterministic scenario, which compensates and justifies the energy requirements for transmitting data packets to the Sink/BS node as shown in Figure 18c and Table 8.

Among the metrics for hierarchical protocols, we took into account the metrics related to node death, included in Table 9.

### 5.5. Other Tests Performed

As a complement to the results obtained, we performed tests with 100, 300, and 400 nodes on areas of proportional size, maintaining the same node density of the 200-nodes tests. These tests were performed in a scenario with random node deployment. Energy assignment for 100, 300, and 400 nodes was also proportional to the 200-nodes tests. For these tests, we only took into account the average values of metrics, using the same metrics of the 200-nodes tests.

In the evaluation of a random scenario with 100, 200, 300, and 400 nodes, the average of CH nodes in the scenario is not relevant. However, the lower variability shown in the variance confirms H-kdtree’s characteristic on-demand CH node selection mechanism, maintaining its variance 83% below LEACH and LEACH-C as shown in the Table 10.

Regarding energy value, results show that H-kdtree maintains energy levels that are very close to those of LEACH, and therefore does not show an improvement in this area. However, H-kdtree shows a significant improvement in QoS as compared to LEACH and LEACH-C as shown in the Table 11.

Table 12 shows the use of an on-demand mechanism implies that the protocol only reacts to a change requested by the network. In the case of H-kdtree’s CH node selection mechanism, this means that it will only be used as a response to receiving a “Death” packet. Note that the number of nodes close to death is the number of CH nodes. Death node in H-kdtree is stepped, and in LEACH and LEACH-C it is incremental.

H-kdtree shows an improvement in delay reduction, with values over 60% as compared to LEACH y LEACH-C, as shown in Table 13.

H-kdtree shows a 95% jitter reduction as compared to LEACH and LEACH-C, as shown in Table 14.

H-kdtree shows a 50% throughput increase as compared to LEACH and LEACH-C, as shown in Table 15.

In the observed metrics in Table 16, show that the stability periods in the half-life of network nodes for the proposed H-kdtree protocol is longer than in LEACH and more short that in LEACH-C. After 50% of the rounds, in H-kdtree, we have found node death to be stepped and maintaining low variability in delay and jitter. This was not only due to its reactive mechanism but also because of its stability derived from using TDMA for medium access. The WSN we studied did not present node mobility: all nodes maintained their positions. This characteristic was used by H-kdtree and its cluster formation mechanism, which is based on the k-d tree algorithm and adds more stability by keeping the majority of nodes in the same clusters after each configuration phase.

The results on PDR showed that TDMA-based packet transmission planning did not show packet loss in LEACH, LEACH-C and H-kdtree, in all scenarios with 100, 200, 300, and 400 nodes.

## 6. Conclusions and Future Work

The H-kdtree protocol has main contributions. First, the clustering formation method based on the k-d tree algorithm partitions the sensor node deployment area in a two-hop hierarchical topology. Second, it is a WSN protocol that provides QoS in support of services with stricter resource demands while keeping energy usage at a level similar to the LEACH protocol.

The proposed H-kdtree protocol was based on the k-d tree algorithm in evaluating the spatial partitioning to organize nodes in a dimensional space (*x* and *y*). The average energy results obtained with LEACH-C, exceed LEACH and H-kdtree on 42%. The partitions found become clusters, creating a network topology that is able to provide QoS for the longest possible time with energy requirements similar to those of LEACH. H-kdtree is characterized by keeping the number of CH nodes stable for the longest number of rounds, maintaining a constant network topology and, as a consequence, low variability in delay, jitter, and throughput metrics. Although these metrics are a function of time, they depend on the variability of the number of CH nodes.

The H-kdtree protocol has three main processes. First, the protocol uses a two-hop network topology that was not altered in each round. Then, during the data transmission phase, the “Death” packet allows H-kdtree to implement a reactive mechanism that only returns to the configuration phase when a node requires it by sending the “Death” packet. This means that the configuration phase is only repeated on-demand. Finally, the minimum group condition allows network traffic to be more homogeneous, which is reflected in delay, jitter, and throughput and, as a consequence, in improved QoS.

The set of experiments performed in random scenarios with 100, 200, 300, and 400 nodes, and a deterministic scenario with 200 nodes, helped us compare LEACH and LEACH-C with the proposed H-kdtree protocol. The conclusion is that the H-kdtree protocol fulfilled the objective by addressing existing problems in cluster generation mechanisms by reducing the variability in CH node formation: with the same resources used in LEACH and LEACH-C, H-kdtree improved delay and jitter by 60% and 95% percent, throughput improved by over 50%, while keeping energy usage at the same levels of LEACH.

Additional experiments will be required to measure H-kdtree’s performance in additional scenarios, incrementing the number of rounds, varying density in environments with heterogeneous node-energy levels, and proposing optimization mechanisms for CH node selection to maximize energy levels in the network. Additionally, with the QoS results obtained, it will be necessary to perform traffic analysis with multimedia data.

## Figures and Tables

**Table 1:** Test data.

	Dimension *x*	Dimension *y*
node 1	54	93
node 2	80	55
node 3	96	86
node 4	74	77
node 5	42	68
node 6	22	45
node 7	11	53
node 8	27	75
node 9	64	29
node 10	81	63

**Table 2:** Simulation parameters.

Parameters	Values
Protocols	LEACH, LEACH-C and H-kdtree
Initial energy	0.5 J
$E_{elec}$	50 nJ/bit
$\epsilon_{amp}$	0.0013 pJ/b/m${}^{4}$
$\epsilon_{fs}$	10 pJ/bit/m${}^{2}$
Data aggregation $E_{DA}$	5 nJ/bit/signal
Message size	4000 bits
Additional energy	α=1
Heterogeneity	m=0.5
CH probability	p=0.3, k=4
Scene	Random, Deterministic
Number of nodes	200
Position node sink/BS (x,y)	(50, 150)
Type of traffic	UDP

**Table 3:** Number of cluster head with 200 nodes. (r) random—(d) deterministic.

Protocol	LEACH (r)	LEACH-C (r)	b	LEACH (d)	LEACH-C (d)	H-kdtree (d)
Min	17	15	23	21	12	20
Q1	24	28	23	27	24	20
Average	28.01	34.15	24.03	30.22	31.69	21.78
Q3	32	40	30	34	39	23
Max	36	68	30	40	74	25
Normalized average	1	1.2192	0.8579	1.0789	1.1313	0.7775
Variance	37.2625	34.8312	2.9384	33.7894	39.7114	5.3248

**Table 4:** Average energy of each node with 200 nodes. (r) random—(d) deterministic.

Protocol	LEACH (r)	LEACH-C (r)	H-kdtree (r)	LEACH (d)	LEACH-C (d)	H-kdtree (d)
Min	0.03918	0.08321	0.04041	0.04849	0.07806	0.04343
Q1	0.05265	0.09902	0.06184	0.06551	0.09443	0.05878
Average	0.07959	0.11681	0.09122	0.09061	0.11377	0.0851
Q3	0.112	0.1343	0.12	0.1188	0.13331	0.1157
Max	0.1416	0.15162	0.142	0.1339	0.1623	0.1414
Normalized average	1	1.4676	1.1461	1.1384	1.4294	1.0692
Variance	0.001139	0.000406	0.001115	0.000951	0.000487	0.001042

**Table 5:** Number of death nodes with 200 nodes. (r) random—(d) deterministic.

Protocol	LEACH (r)	LEACH-C (r)	H-kdtree (r)	LEACH (d)	LEACH-C (d)	H-kdtree (d)
Min	0	0	0	0	0	0
Q1	1.941	0	0	0	0	0
Average	27.06	13.96	9.7799	13.52	6.91	10.38
Q3	48.29	25	10.012	22.5	12.5	16
Max	76	68	78	44	33	27
Normalized average	1	0.5158	0.3614	0.4996	0.2553	0.3835
Variance	677.3701	401.614	368.7591	232.9591	106.38	120.3591

**Table 6:** Delay with 200 nodes. (r) random—(d) deterministic.

Protocol	LEACH (r)	LEACH-C (r)	H-kdtree (r)	LEACH (d)	LEACH-C (d)	H-kdtree (d)
Min	0.1568	0.077	0.02986	0.13	0.039	0.01
Q1	0.4328	0.573	0.09687	0.4273	0.5565	0.02026
Average	0.928	0.8758	0.114	0.8495	0.9068	0.04285
Q3	1.324	1.1715	0.1392	1.236	1.3115	0.04935
Max	1.588	1.56	0.1411	1.546	1.669	0.1094
Normalized average	1	0.94375	0.1228	0.9154	0.9771	0.0461
Variance	0.251437	0.16277	0.000960	0.218075	0.21478	0.000851

**Table 7:** Jitter with 200 nodes. (r) random—(d) deterministic. The values of the table are on a scale of $10^{-3}$.

Protocol	LEACH (r)	LEACH-C (r)	H-kdtree (r)	LEACH (d)	LEACH-C (d)	H-kdtree (d)
Min	7.478	6.88	4.611	7.652	6.04	4.988
Q1	11.47	21.3	4.611	8.928	48.12	4.988
Average	20.5	40.61	4.815	21.46	33.77	5.25
Q3	22.07	57.88	5.05	25.64	48.12	5.53
Max	55.46	87.03	5.05	61.5	79.84	5.635
Normalized average	1	1.9809	0.2348	0.10468	1.6473	0.25609
Variance	0.2185	0.55136	0.00003998	0.4729	0.35932	0.00006838

**Table 8:** Throughput with 200 nodes. (r) random—(d) deterministic.

Protocol	LEACH (r)	LEACH-C (r)	H-kdtree (r)	LEACH (d)	LEACH-C (d)	H-kdtree (d)
Min	22,630	20,267	140,400	12,060	5096	133,200
Q1	66,410	65,741	145,900	40,210	49,350	135,100
Average	97,940	107,854	145,900	104,300	101,705	136,500
Q3	131,900	149,547	150,300	154,400	149,510	137,600
Max	178,200	190,903	150,300	172,200	192,501	141,500
Normalized average	1	1.0122	1.4896	1.0649	1.0384	1.3937
Variance $\times 10^{6}$	2154.20	2285.91	9.49	3502.15	3346.94	12.3741

**Table 9:** Comparison of network lifetime with respect to FND, HND and LND with 200 nodes. (r) random—(d) deterministic.

Protocol	LEACH (r)	LEACH-C (r)	H-kdtree (r)	LEACH (d)	LEACH-C (d)	H-kdtree (d)
FND	24	47	38	37	51	35
HND	113	99	94	122	103	96
LND	134	164	131	144	180	140

**Table 10:** Number of cluster head. (r) random.

Number of Nodes		LEACH (r)	LEACH-C (r)	H-kdtree (r)
100	Average	29.34	13.703	28.16
	Variance	42.4871	34.0309	4.7477
200	Average	28.01	34.15	24.03
	Variance	37.2625	34.8312	2.9384
300	Average	24.80	43.4356	32.43
	Variance	39.3804	48.0083	6.4618
400	Average	34.11	39.5644	32.67
	Variance	46.3957	39.7683	5.6382

**Table 11:** Average energy of each node. (r) random.

Number of Nodes	LEACH (r)	LEACH-C (r)	H-kdtree (r)
100	0.06682	0.0901	0.07915
200	0.07959	0.11681	0.09122
300	0.08839	0.1217	0.09623
400	0.09272	0.1433	0.1062

**Table 12:** Number of death nodes. (r) random.

Number of Nodes	LEACH (r)	LEACH-C (r)	H-kdtree (r)
100	12.35	5.3465	6.39
200	27.06	13.96	9.77
300	43.08	21.9604	32.83
400	64.72	33.521	58.91

**Table 13:** Delay (seg). (r) random.

Number of Nodes	LEACH (r)	LEACH-C (r)	H-kdtree (r)
100	0.283324844	0.8021	0.10480334
200	0.928	0.8758	0.114
300	0.841188642	0.6348	0.177976
400	0.6150795862	0.9217	0.104979

**Table 14:** Jitter (seg). (r) random.

Number of Nodes	LEACH (r)	LEACH-C (r)	H-kdtree (r)
100	0.0200929664	0.2216	0.00499735
200	0.0205	0.04061	0.004815
300	0.0227276422	0.03975	0.00462749
400	0.0200274796	0.2368	0.00552575

**Table 15:** Throughput (bps). (r) random.

Number of Nodes	LEACH (r)	LEACH-C (r)	H-kdtree (r)
100	96,134	101,319	135,210
200	97,940	107,854	145,900
300	102,603	110,623	163,092
400	90,956	96,521	170,688

**Table 16:** Metrics of network lifetime. (r) random.

Number of Nodes		LEACH (r)	LEACH-C (r)	H-kdtree (r)
	FND	26	36	30
100	HND	121	88	83
	LND	147	184	127
	FND	24	47	38
200	HND	113	99	94
	LND	134	164	131
	FND	39	71	45
300	HND	119	117	89
	LND	152	174	149
	FND	52	115	37
400	HND	137	154	101
	LND	164	223	152

## Abbreviations

The following abbreviations are used in this manuscript:

CH	Cluster Head
WSN	Wireless Sensor Networks
IoT	Internet of Things
CPU	Central Processing Unit
LEACH	Low Energy Adaptive Clustering Hierarchy
LEACH-C	Low Energy Adaptive Clustering Hierarchy-centralized
QoS	Quality of Service
PDR	Packet Drop Rate
BS	Base Station
GPS	Global Positioning System
RSSI	Received Signal Strength Indicator
TDMA	Time Division Multiple Access
HEED	Hybrid. Energy-Efficient Distributed
TEEN	Threshold sensitive Energy Efficient sensor Network
NS-2	Network Simulator
EED	End-to-End Delay
FND	First Node Died
HND	Half of Nodes Died
LND	Last Node Died
H-kdtree	Hierarchy protocol based on the k-d tree algorithm
